# Challenges in health service delivery under public-private partnership in Tanzania: stakeholders’ views from Dar es Salaam region

**DOI:** 10.1186/s12913-020-05638-z

**Published:** 2020-08-18

**Authors:** Said Nuhu, Chakupewa Joseph Mpambije, Kinamhala Ngussa

**Affiliations:** 1 Institute of Human Settlements Studies, Ardhi University, Dar es Salaam, Tanzania; 2 Department of Urban and Rural Development, Swedish University of Agricultural Sciences, Uppsala, Sweden; 3 Department of Development Studies, History and Political Sciences, Mkwawa University College of Education (MUCE), Iringa, Tanzania; 4 Institute of Development Studies (IDS), University of Dar es Salaam, Dar es Salaam, Tanzania; 5 College of Business Education (CBE), Dar es Salaam, Tanzania

**Keywords:** Public-private partnership, Institutional arrangement, Health service delivery, Stakeholders, Tanzania

## Abstract

**Background:**

Public-private partnership in the health sector was introduced to improve the delivery of health services in Tanzania. Contrary, the expected outcomes have not been fully realised. This study aimed at investigating challenges encountered in implementing public-private partnership institutional arrangements in health service delivery in Kinondoni Municipality, Dar es Salaam, Tanzania.

**Methods:**

A qualitative case study design was employed, where in-depth interviews with stakeholders were held and document reviews conducted. Fourteen (*n = 14*) participants engaged in this study. Eight (*n = 8*) and six (*n = 6*) of the fourteen participants were from the public and private sector respectively. The thematic approach was used to analyse data, and ethical principles in the research process were upheld.

**Results:**

Findings revealed that although public-private partnerships are hailed for supplementing the government’s efforts in the provision of health services, institutional arrangements for the smooth provision of these services are lacking. Several challenges encumber smooth provision of health services and these include inadequate resources, ineffective monitoring and evaluation, and insufficient consultations between partners.

**Conclusion:**

Inadequate legal and policy framework, or ineffective implementation practices may influence challenges facing institutional arrangements for public-private partnerships. Therefore, strengthening of public-private partnerships is recommended to improve implementation mechanisms and practices such as adherence to partnership agreements and compliance to the policies, laws and regulations.

## Background

Public-private partnerships were introduced to supplement the government efforts in the provision of health services in Tanzania. Comprehensive Health Sector Reforms (HSR) as policy responses to the Structural Adjustment Programmes (SAPs) were implemented in the 1990s [1, 2]. These reforms included privatisation of health services and introduction of: user fees; pre-paid system; and health insurance schemes [1, 3]. Public-Private Partnership (PPP) also emerged from these reforms as an institutional arrangement between the public and the private sector [1, 2, 4]. In this study, PPP is defined as the collaboration between government agencies and the private sector for the purpose of delivering health services. The introduction of PPP aimed at improving the quality of health services and expanding access of these services in the community; increasing opportunities for the private sector to invest in healthcare; and formalising non-profit organisations to engage in healthcare service provision [2, 4, 5]. Significantly, it was also aimed at enhancing efficiency and effectiveness of health service delivery to achieve value-for-money, encourage innovation and develop more user-sensitive services appropriate for a particular context [6]. The introduction of PPP necessitated the development of a supportive regulatory framework in Tanzania to ensure effectiveness and efficiency in the delivery of health services.

Various legal and policy guidelines have been developed to support implementation of PPP. Effectiveness of PPP in health service delivery is predicated on the nature of a particular institutional framework (formal and informal rules as well as constrains), which provide guidelines for implementation [7, 8]. The rules define private property rights and their allocation; the relationship between participants in the partnership; conventions governing participation such as competition, fair business practices and mutual trust [8, 9]. In this regard, the government established policies and regulations such as the PPP policy of 2009 and the PPP regulation of 2011. PPP is also guided by the Tanzania Investment Act of 1997, the Public Procurement Act of 2011, and the Public-Private Partnership (Amendment) Act of 2014 [1, 10–14]. The guidelines in the legal and policy documents are aimed at strengthening partnerships to support healthcare service delivery and accessibility. To ensure this, the government of Tanzania has adopted three modes of health service delivery under PPP, namely; contracting out, offering subsidies and privatisation.

Health services delivery modes have varying contractual specifications. Under the contracting out mode, the government provides all necessary infrastructures, human and financial resources which are handed over to the private health entrepreneur(s). Regarding subsidies, the government provides some incentives and financial assistance to private health hospitals to facilitate the provision of specific health services such as maternal and child healthcare. Within the privatisation mode, the government either surrenders its health facilities and infrastructures to private individuals/institutions, or allows the latter to develop their own infrastructures, and the government offers subsidies in terms of tax and supplies [15]. The existence of different modes of partnerships creates room for several healthcare opportunities for the private sector.

Regardless of the mode of health service delivery, PPP arrangements have several benefits. The establishment and implementation of PPP in the health sector in Tanzania has contributed to the reduction of challenges associated with health services delivery and accessibility of preventive and curative measures [4]. Additionally, the reduction of maternal and infant mortality for example can also be attributed not only to government-owned facilities but also to the existing private health facilities in partnership with the government [16]. It has also facilitated accessibility of health services in remote areas, contributed to reduction of emergency cases at public hospitals, led to the improvement of health service delivery because of competition and provided employment opportunities [17]. The significance of PPP in the health sector has thus attracted a debate among scholars.

Extensive studies have been conducted in the thematic areas of PPP, in the health sector in Tanzania and in other countries. There seems to be a consensus on the deterioration in the health sector and support for the participation of the private sector under PPP as a critical solution [1, 18, 19]. Studies show that PPP has enabled: access to health services in terms of distance; affordability in terms of reduction of health costs; and improvement in the quality of health service delivery [2, 20–22]. However, PPP arrangements have not been without challenges. A review study by Basu et al. on the performance of PPP in low and middle-income countries and Whyle and Olivier in Southern Africa revealed a violation of medical standards and inadequate: human resource; equipment; and medication in the public sector [23, 24]. Notably, different geographical settings may have varying contextual challenges and therefore the findings from Basu et al., Whyle and Olivier may not universally apply in all low and middle-income countries. Other studies observe that PPP in general but particularly in the reproductive and child health services has encountered challenges regarding informal partnerships and weak governing structures [4, 25]. Some studies have focused on contractual arrangements between faith-based institutions providing health services and the government [26, 27]. These disparities and challenges in faith-based institutions may not apply to secular private health facilities owned by individuals, or other companies because of differences in business orientation and governance structures.

The evidence reviewed focused on benefits of PPP in the delivery of health services, challenges within a specific health focus, while some studies had methodological limitations. Few studies have articulated the nature of PPP arrangement and operation in Tanzania, as well as its implication for effective delivery of health services [20, 28]. The aim of this study is to investigate challenges of PPP arrangements in health service delivery based on stakeholders’ views and experiences in Kinondoni Municipality, Dar es Salaam, Tanzania. The study offers some critical insights into the health sector and PPP in particular. It also contributes to the growing body of literature in public health.

## Methods

### Study design and sites

A qualitative case study design was used to allow a deeper insight into the PPP phenomenon in health service delivery. The design provided an opportunity to collect data related to experience, meaning and perspectives from the stakeholders’ point of view as firsthand participants [29, 30]. Views captured were in regard to PPP institutional arrangements and existing challenges. Kinondoni Municipality in Dar es Salaam was selected based on the following three criteria: (i) complex, heterogeneous and densely populated areas; (ii) high concentration of healthcare facilities in PPP arrangements; (iii) facilities reported having PPP implementation challenges. Within Kinondoni Municipality, the study focused on four wards, namely: Kijitonyama, Magomeni, Mikocheni and Sinza because these wards are densely populated. The wards have a variety of healthcare infrastructure which includes both public and private healthcare facilities.

### Selection of health facilities

The criteria for selecting health facilities was based upon; (i) health facilities offering both public and private services; (ii) and facilities with PPP institutional arrangement challenges. This was an informed decision guided by the information from reviewed documents from the Ministry of Health, Community Development, Gender, Elderly and Children (MoHCDGEC)- the former Ministry of Health and Social Welfare, as well as information received from health officials at the region and district. Thus, three hospitals namely: Sinza Government Hospital, Marie Stopes private hospital and Herbert Kairuki private hospital were sampled. Two health centres; Mama Ngoma Health Centre (private), Magomeni Health Centre (government), and three dispensaries; Mwenge Dispensary (government), Arafa Capricon Dispensary (private) and MicoSinza Dispensary (private) were also sampled.

### Study participants

Fourteen (14) participants in this study were purposefully selected from the MoHCDGEC, local government authorities, public and private health centers, dispensary or hospitals with PPP arrangements. Hence, two (*n = 2*) officials from the MoHCDGEC, three officials (*n = 3*) from Kinondoni municipality, three officials (*n = 3*) from public health facilities and six officials (*n = 6*) from private health facilities took part in the study. Officials from the MoHCDGEC and the District health department were selected by virtue of their responsibilities and experience in implementing PPP, as indicated in Table 1. Representatives of the Medical management team from both the private and health facilities were selected as administrative focal persons from sampled health facilities.

**Table 1:** Participants characteristics

Category	Participants	No. of interview	Responsibility	Experience
Government	MoHCDGEC	2	Formulating, reviewing, implementing and monitoring of the Ministry’s policies.	10+ years
	District Health Office	3	Implementation and monitoring of health programmes within the district and providing guidance and assistance to personnel. Liaising with other district authorities, higher authorities in the health sector and also performing other administrative duties in the district. Planning and budgeting for health service delivery in the district; mobilising financial, medical and human resources. Preparing health development plans on the basis of national policies and direction.	6+
	Health facilities team	3	Overall supervision of health services delivered at facilities and supervision of staff. Ensuring smooth implementation and mainstreaming of government programs at the facilities and in the communities. Preparing budgets, ensuring safe custody of medical supplies and equipment, as well as delivering professional medical services.	5+
Private	Hospital management team	3	Preparing budgets and supervising the procurement process. Ensuring safe custody of medical supplies and equipment. Coordination with stakeholders. General administration and discipline of staff. Conducting periodic progress and review exercises.	4+
	Health centers management team	3	Providing and maintaining up to date inventory of facilities and equipment. Ensuring efficient and effective delivery of services. Supervising health education of patients and the community around. Making proper diagnosis of diseases, prescribe treatment, treat minor injuries, attend general outpatient clinics. Supervising and monitoring staff performance and activities.	4+
Total		14		

### Data collection methods

In-depth interviews with participants and document reviews were conducted to collect data. The interviews enabled collection of detailed information regarding institutional arrangements in health service delivery. An interview guide was developed for this study and is provided as additional File 1. The guide was used to collect data on issues focusing on roles and power of partners in decision making (planning, budgeting and implementation) under PPP. It also explored implementation of PPP institutional arrangements, as per the regulatory framework. Interviews were conducted in 2017 and 2018. Additional information from document reviews was added in 2019. Participants in interviews were engaged for thirty (30) minutes to one (1) hour at the participants’ place of work, with a prior appointment for the scheduled time. These interviews were recorded using a tape recorder with consent from the participants.

Documented evidence was collected and examined to elicit meaning and gain understanding of existing challenges in PPP institutional arrangements. The reviewed documents included the Comprehensive Council Health Plan (CCHP) for Kinondoni District of 2015 and 2016, which provided information on the status of the health sector regarding the number of private and public health facilities, financial information, health infrastructure development, among others. Policies and laws reviewed included the National Public Private Partnership Policy endorsed in 2009 and the Public Private Partnership Regulation of 2015. Other reviewed documents were the Health Sector and Social Welfare public-private partnerships policy guideline of 2011 and the National Health Policy of 2007. Among other things, the policy guidelines were also reviewed to establish the mandated duties and responsibilities of the partners under PPP, as well as modes of partnerships and implications.

### Data analysis

The researcher(s) applied a thematic approach to analyse the study findings. Tape-recorded in-depth interviews were transcribed verbatim and translated from Swahili into English. At the end of each day, transcribed field notes were read several times to identify common patterns. These were crosschecked with tape-recorded information to include what the author(s) could have missed to note down. Common ideas that emerged included inadequate funds, late disbursement of insurance funds, poor budget allocations, high operational costs, unskilled PPP implementation team, inadequate staff, insufficient logistics and supplies, lack of transparency, bureaucracy, poor accountability mechanisms, lack of adherence, and ineffective policies, among others. These common ideas were coded and categorised into common themes which include; existing PPP health facilities, institutional arrangements and challenges as presented in Section 3 (result section). Data from reviewed documents were analysed according to themes of the study to complement data got from in-depth interviews.

### Ethical issues

Prior to data collection, participants were briefed about the purpose of the study, to get informed consent and voluntary participation. The consent for participation was verbal because the study wanted to protect the job security of participants, since some views would blame the partners. The university was mindful of this decision before releasing the researcher to go to the field for data collection. Thus, Ardhi University provided the research clearance letter, Number ARU/ARU/HO 67/VI, on behalf of the government and the Tanzania Commission for Science and Technology (COSTECH). The regional and district authorities in their respective administrative units also approved the research to be conducted in the study area.

## Results

This section presents empirical findings based on three broad categories. The first sub-section presents the number and nature of health facilities found in Kinondoni municipality, the second sub-section presents the guiding institutional arrangements for PPP operations and the third sub-section presents obstacles existing in the implementation of PPP operations.

### Public and private health facilities in Kinondoni municipality

**Fig. 1:**
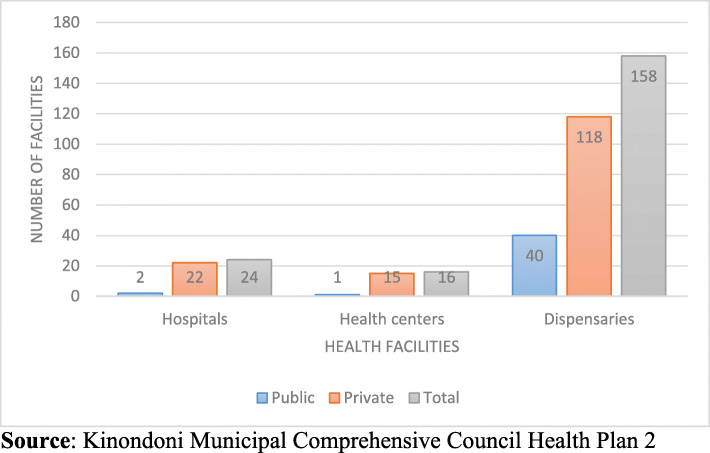
Distribution of Public and Private Health Facilities in the Kinondoni Municipality

The number of health facilities in the municipality and specifically under the PPP arrangement reveals the quantity and quality of health service provision in the area. The Comprehensive Council Health Plan (CCHP) of 2016 reveals that Kinondoni municipality has more private health facilities than public. Kinondoni Municipality has 24 hospitals, 16 health centres and 158 dispensaries. Findings indicate that 78% are private health facilities, while only 22% are public. The municipality has 22 hospitals, 15 health facilities and 118 dispensaries, which are privately owned, while 2 hospitals, 1 health centre and 40 dispensaries are public facilities as shown in Fig. 1.

The existence of more private health facilities than public ones does not contravene the Tanzania National Health Policy of 2007 and the Public Health Policy Act of 2010 which state that where there is a private health facility, the government should not construct another health facility of the same nature. Figure 1 reveals that the private sector in Kinondoni municipality responded positively to the national call by government for the private sector to engage in health service provision. Basing on the current statistical trends and policy guidelines as revealed, the private sector may continue to dominate the district in terms of numbers of health facilities and the number of users of health services in these facilities. Findings revealed that existing facilities operating under PPP provide different clinical services that were previously predominantly provided by the public sector. These include provision of clinical services to pregnant mothers, vaccination of children under 5 years, and inclusion of the national health insurance scheme for service users.

### Institutional arrangements for provision of health services under PPP

The nature of institutional arrangements under PPP is critical to understand implementation flaws. Documents reviewed indicate that provision of health services in Tanzania and Kinondoni municipality in particular, follow the established institutional arrangements that guide PPP implementation. Interviews with health officials from the municipality revealed that the existing public and private actors in health service provision are bound by the established institutional arrangements. Findings established that contracting mechanisms in PPP, marked the establishing of institutional arrangements in Kinondoni municipality. The municipality entered contracts and service agreements with private health care providers guiding the delivery of health services. Key performance indicators were established in aspects of performance management, output measurement and management reporting to enable a robust assessment of contracted performance.

It was revealed that establishing collaboration in decision making and information flow among PPP actors was an important institutional arrangement to facilitate the implementation of PPP processes. Interviews with participants attested that each partner agreed to form an entity body that could ensure that decisions are made in agreement with both parties. Under the public sector, different bodies were established to ensure transparency in decision making and smooth flow of information. These included establishment of the Council Health Management Team (CHMT), the District Health Planning Team, the Council Health Service Board (CHSB) and the Health Facility Governing Committees (HFGCs).

Finding from reports indicated that another institutional arrangement was provision of incentives to the private health facilities. This was to encourage result-based services, to strengthen partnerships with private entities. It was revealed that the government through the municipality provides or supports human resource capacity building in private health facilities, particularly for-non-profit healthcare facilities. For instance, the municipality provides health workers to MICO Rabininsia Memorial Hospital and Mbweni Mission Hospital for delivery of services such as Maternal and Child Health (MCH) care, vaccinations and provision of care and treatment clinic to Tuberculosis (TB) infected persons and people living with HIV/AIDS. Also, the private health facilities are provided with medicines such as ARVs and medical equipment. The municipality ensures that partners receive the incentives expected in accordance with the district annual budget. Reiterating this, one official explained that:

“The municipality allocates ten percent of the budget annually for private health service facilities. In the past, we were allocating these incentives in terms of financial resources only, but recently we have also provided medical equipment, medicines and human resource support.”

The official from the MoHCDGEC gave more clarification concerning incentives. Private health facilities which comply and meet the set standards in the institutional arrangement are prioritised for supplies of incentives to meet the technical or financial needs.

### Obstacles to health services delivery under PPP

In this study, several challenges that constrain smooth implementation of PPP arrangements in the delivery of health service in Kinondoni municipality were revealed. These are categorised into: regulatory issues, inadequate resources, ineffective monitoring and evaluation of PPP activities as well as insufficient consultation and communication.

### Regulatory issues

The study reveals that the PPP policy of 2009 was built upon structures already established under the Health Sector Reforms (HSRs). Therefore, the policy may not adequately address the emerging challenges under PPP. Notably, the study reveals implementation challenges attributed to inadequacies in the guidelines. It was observed, for instance, that the PPP governance structures at local level were lacking and therefore implementation of the PPP activities was overseen by the MoHCDGEC. This creates bureaucratic governance issues whereby sometimes partners at the local level are answerable to the highest office. Local Government Authorities (LGAs) under this arrangement are contractual authorities with a budget and responsibility to build the capacity of personnel to implement PPP health service delivery. However, this has not been effectively implemented as one participant revealed.

“ … as local government, we have not been able to train the staff in the health department engaged in PPP activities...that is why in most cases, it is the line ministry officials active in implementing the PPP activities in the district”

The revelation reflects the bureaucratic governance mechanisms witnessed in implementing PPP activities in the district and lack of skilled personnel to oversee PPP arrangements’, which are attributed to policy inadequacies. Besides inadequate guidelines, the study also reveals a lack of compliance to the existing policies and established guidelines. The municipality through the District Medical Office (DMO) enforces regulations and standards guiding healthcare provision and ensures adherence to the professional conduct of ethics. To ensure universal access to healthcare service for all, the municipality has put in place regulations to guide both public and private healthcare providers. This includes treatment of patients in emergency cases, regardless of their ability to pay for the services. It was revealed that this policy has not been upheld because in some health facilities, health service providers establish their own prices, most of which were too costly for the majority of the vulnerable poor. In an interview with one official from the private health facility, the following words were echoed:

“ … in the first place, this facility was established for income generation and therefore we have standard service charges for specific cases and if the facility has to be sustained, charges must be levied on every client regardless of economic or health status. It is also costly to handle some emergence cases.”

The official further expressed fear that some patients could use this opportunity to seek for free services in the name of emergence. It was also expressed that if the government would supplement the facility with adequate resources, this guideline would be implemented without failure to comply. The PPP policy guideline of 2013 also shows that partners need to participate and agree upon all matters related to budgets and other plans. Officials from private health facilities lamented that decisions are drawn by the government (top-down approach). Often the decision-making process has not been participatory. For instance, the private health service providers noted lack of transparency in the guidelines for the CCHP, especially in the allocation of funds within the budget framework, hence hindering effective implementation of the service agreement.

It was also noted that private health facilities were by-passing the established hierarchy of referral systems that is emphasised by the MoHCDGEC. This was depicted by the tendency of private dispensaries and private health centres referring patients directly to more specialised hospital levels like Mwananyamala Hospital (government), Herbert Kairuki Hospital (private) and Muhimbili National Hospital (government), as revealed by an official from one of the private health facilities:

“ … sometimes we do not follow the hierarchy for referral management and we just refer patients to hospitals like Sinza Hospital, Mwananyamala Hospital, or any private hospital where we are sure that the client will receive specialised treatment.”

The official further revealed that they did not offer official letters to clients referred to other healthcare facilities without following the established referral hierarchy. This reveals lack of coordination between healthcare facilities and lack of clear follow-up plans. It also shows a lack of formal managerial meetings between private health facilities on one hand and other established bodies like CHMT, CHSB, HFGCs at the district with the responsibility to oversee smooth implementation of PPP regulations. Far from the regulatory framework being inadequate and ineffective, financial constraints may also act as barriers to effective PPP arrangements.

### Inadequate resources

Lamentably, in the implementation of the PPP policy, practice and monitoring of activities was paralysed by inadequate resources. The government has not been able to deliver on its promises in the PPP arrangement on key issues, especially provision of financial support and requirements. This has affected provision of health services using the National Health Insurance Fund (NHIF) payments because of the delays in disbursement of funds by the government as one official from the health facility management team revealed:

“ … clients cannot use the NHIF card in our health facility because the government has not yet disbursed funds for this scheme … we are only serving clients able to pay.”

This facility was not accepting to treat patients enrolled under the national insurance scheme. However, in other private facilities, it was observed that patients were using the insurance identification cards and were being offered services. When the health officials were probed on this, they mentioned that they treat the patients even when the government has not deposited the funds on the account, because of the realisation that if patients were to be turned away, most people would not be able to access health services at the facility as one official confided:

“ … you see … we cannot wait for the government to first put money on the fund account to serve clients under the scheme because normally the government delays to effect disbursement of funds... so in this facility we opt to save lives first as we wait for the money”

Revelations show that private facilities hence have to invest in this partnership because if they were relying on government’s support, they would not deliver health services to all. It was revealed that when the government delays to disburse funds, this affects fulfillment of the partnership and may lead to facilities turning away patients under the national insurance scheme. The delay in disbursement of funds is not only a concern between the local government and health facilities but also between local governments and the MoHCDGEC as well. Inadequate resources was also noted as hindering effective information flow in the decentralisation of health service delivery. Therefore, most of the health providers in Kinondoni municipality did not have the capacity to respond to collaboration needs.

### Lack of trust between PPP partners

Participants revealed the problem of trust between implementers of PPP arrangements in Kinondoni municipality. Whereas private health providers blamed the existing bodies responsible for overseeing the implementation of the PPP activities in the municipality, they also blamed the private health service providers for not fulfilling the decisions agreed upon. For instance, private health service providers kept charging high service fees contrary to the service agreement. Furthering this argument, one official from the municipality commented:

“ … the goal of private health facilities is to maximise profit and therefore sometimes these facilities do not adhere to service agreements. When we make impromptu supervisions, we find the health facilities operating contrary to the agreed upon decisions.”

Private health service providers expressed their disappointment for not being equally represented in the decision-making bodies at the district. Although private healthcare facilities were involved in some decision-making processes in the CHSB, CHMT, District Health Planning Team, and the Hospital Governing Committee, it was difficult to have influence and power over the district’s decisions on health interventions. The private healthcare facilities feel overpowered by government bodies and therefore do not trust that the decisions reached may be in their favour. Such revelations from the implementers of PPP in Kinondoni municipality portray challenges posed by lack of trust and its implication for health services delivery.

### Ineffective monitoring and evaluation of PPP activities

Successful implementation of PPP largely depends on effective monitoring and evaluation of the agreed performance indicators between partners as developed by the MoHCDGEC. These include the degree of collaboration between partners in terms of numbers, contribution to the partnership and client satisfaction rate. These performance indicators were used in monitoring and evaluating the performance of health service providers at the municipal level as revealed by one of the health officials that:

“Our district has designed monitoring and evaluation mechanisms which demand partner adherence. We always evaluate how partners follow agreed-upon procedures in all implemented activities to make informed decisions.”

This shows that the government has been keen on monitoring to ensure efficiency and effectiveness of health service delivery under PPP. Overall, the central government is mandated to periodically monitor and supervise activities in line with PPP service agreements with partners. In line with the decentralisation guidelines, primary health service providers are supposed to be monitored by the LGAs, while regional hospitals are under the supervision of regional authorities. The discussion with government officials revealed that reports from partners were sometimes not submitted, or not written, and hence the monitoring team lacked a foundation to assess progress. Showing concern, the official from government lamented:

“ … It is always assumed that the government is not serious, but even the private sector is not effective … Imagine, sometimes the private health facilities under PPP do not submit periodic reports but they need financial support...how do they expect the government to take action when there is no evidence to show activities conducted and services provided?”

Monitoring and evaluation of the PPP activities have not been adequately conducted since there is missing information and lack of compliance by private partners. It was also revealed that enforcement of compliance was ineffective because of limited funds. The district health officials consulted admitted that supervision costs are very high for the municipality and therefore adequate supervision is hard to effect.

### Insufficient consultation and communication

Private health facilities are mandated to send representatives to the respective bodies, namely CHMT, HDPT, CHSB and HFGCs, among others at the district to partake in the implementation of different PPP processes and activities. Findings revealed however that these representatives were not always consulted. The interview with one official from a private health facility revealed weaknesses in the consultation accountability processes. The process of referral procedures for instance from a private facility to a government facility is affected by poor consultation between the partners. Administrative officials from private health facilities noted that the planning approach is bottom-up and allows for involvement of the private sector, however, they are not consulted adequately during the planning process. Hence, participation in planning, budgeting and management of resources remained the authority of the Council for Health Management Team at the district, instead of being done collaboratively with the private sector. During the interview with one official from the private health facility, it was asserted that:

“ … when the Ministry of Health introduced Big Result Now (BRN) plan for implementation, for the purpose of attaining the Development vision 2025, Partners were never consulted, hence our ideas were not incorporated in this plan … the realisation of the vision is thus doubted”

This revelation depicts lack of consultation with the private sector in the health service provision and yet, such a strategy requires the participation of key stakeholders in the planning process for smooth implementation. The private sector hence feels marginalised in the partnership with the public sector, especially at the national and local government level, where insufficient communication flows over initiatives meant to strengthen PPP are undertaken.

The provision of health services under PPP in Tanzania has faced significant challenges accruing from inadequacies in the institutional arrangements for implementation of PPP activities. These may be associated with the public sector, or private sector. The study has also revealed inadequate regulatory mechanisms, as well as non-compliance issues.

## Discussion

The paper aimed at unravelling existing challenges in the PPP institutional arrangement in the delivery of health services in Tanzania. In exploring these challenges, interviews with relevant officials were conducted and relevant documents reviewed. Information generated focused on implementation and obstacles to smooth operation of PPP institutional arrangements. Obstacles highlighted focused on regulatory issues, resources, monitoring and evaluation mechanisms and outcomes, consultative decision making and communication.

The delivery of health services in Kinondoni municipality hinges on the implemented PPP institutional arrangement. There are more private health facilities than public. The common mode of PPP institutional arrangement in the municipality is the contracting mechanism that involves establishment of service agreements between partners. Decision-making procedures and information flow, as well as provision of incentives to private health facilities as enshrined within the institutional arrangement, operates. However, some challenges exist in the implementation of PPP activities.

One of the significant limiting factors for effective implementation of PPP agreements is inadequate resources. The problem of disbursing financial resources through the National Health Insurance Fund (NHIF) for instance emerged as a critical hindsight. This has been evidenced to be a perennial not only in Kinondoni municipality but also countrywide, as evidenced that the government had delayed to disburse funds to faith-based hospitals in four districts [26, 27]. The situation in Tanzania is like some African countries which fail to disburse funds in time, or where funds are not distributed at all, impending the delivery of services [31]. Countries like Chad, Uganda, and Cameroon do not allocate funds as agreed [31]. Notably, funding is critical for the sustainability of the contractual relationship between the government and partners for meaningful PPP arrangements. Therefore, failure to support the private sector facilities hinders the success of PPP institutional arrangements in poor countries. Inadequate resources hinder the implementation of other activities that ensure smooth implementation of PPP arrangements such as monitoring and evaluation, among others.

It is critical for private healthcare facilities under PPP establishments to be monitored to ensure compliance to contractual agreements for effective and efficient delivery of health services. Monitoring and evaluation of PPP activities is mandatory according to the Tanzanian National Public Private Partnership Policy of 2009 [32]. The policy states that the government in collaboration with the private sector will: (i) prepare a monitoring and evaluation framework, including performance indicators and benchmarks; (ii) set up a timeframe for evaluation; (iii) and review the policy and associated legislation as when need arises. However, the execution of PPP is not monitored and evaluated adequately according to policy requirements and indicators as revealed. Similar challenges have been highlighted by other scholars [24, 33]. Raman and Bjorkman concluded that the problem of ineffective monitoring and evaluation of PPP in the health sector emanates from paying less attention to performance indicators [33]. Basu argues that the problem of monitoring and evaluation of PPP is exacerbated because the government does not have adequate capacity to monitor and evaluate the private sector [24]. These observations suggest that, to a large extent, enforcement procedures could be weak, accountability mechanisms lacking and therefore partners become reluctant in implementing PPP activities. Poor monitoring and evaluation procedures may also indicate poor communication structures and processes.

Barnes argues that effective PPP needs to be incorporated with a clear taxonomy of effective communication that helps practitioners to design successful implementation plans and establish realistic expectations [34]. This was also confirmed in the present study as ineffective information flow and communication sharing adversely affected smooth implementation of PPP activities in Kinondoni municipality. Similar findings have been evidenced from other studies, particularly from developing countries such as Ghana, Zimbabwe and Kenya [24, 35]. Importantly, a good communication plan can facilitate mutual engagement and participation, which may create a level of trust that is required for PPP to succeed. Effective communication also helps to overcome bureaucratic hostility, which in the long run increases transparency in managing PPP related matters in the health sector [36]. Communication can pave the path for a two-way dialogue on contentious issues before the public confidence and trust erode.

The regulatory framework guiding the implementation of PPP arrangements sometimes gives more power to the public sector. Mahoney and Thelen note that institutional arrangements are important in determining power relations, which determine partners’ decision making [37]. Existing power relations between partners while implementing PPP activities may trigger formidable challenges when each partner struggles to maintain power. Belt and Spierenburg caution that in implementing PPP, the distribution of benefits or outcomes should reflect upon power relations [38]. In partnership, power may be exercised based on coercion, either politically or financially, besides the authority and legitimacy [39]. In Kinondoni municipality, the government, because of her power and authority, has been influencing decisions in implementation of PPP plans. The private health facility owners have been given little room to make decisions in the partnership while delivering health services in the municipality. The existing PPP institutional arrangement in Kinondoni reflects what Buse and Harmer have attested that the central government and local government have more power to make decisions at the expense of the private health facility owners [39]. Notably, the existing relations have never been a neutral tool that may realise a win-win situation for all partners involved. Sevilla emphasises that the specific format of PPP, in any situation, depends on the policy and regulatory framework, which often needs to be adjusted to accommodate new types of institutional arrangements, partnerships and collaborations [40].

PPP arrangement is more affected by weak governance mechanisms. These include inadequate policies, poor enforcement mechanisms, lack of transparency and unequal participation in decision making [4, 41]. Efficiency of PPP performance in health service provision is also influenced by divergent partners’ missions, strategies and values [42]. Whereas, the primary aim of the public sector is to improve access to health services, the private sector is profit oriented [24, 42]. While PPP is expected to supposedly improve the quality of healthcare service, sometimes this is compromised in the spirit of maximising profits [24, 43]. Despite these challenges, PPP can be improved through careful crafting of agreements and negotiations, as well as enabling partners to cooperate and fulfil their interests and goals.

This study is not without strengths and limitations. The strength of this study lies in the fact that it has the ability of assisting health policy makers in the country with evidence on the persistent challenges that still face PPP institutional arrangements. These findings also contribute ideas towards an endless debate about the benefits and challenges of PPP in the health services delivery in the context of Africa countries. Different stakeholders namely health service providers, communities and implementers of the existing partnerships will find this study useful as it has shed light on the challenges. This study reveals views from stakeholders with diverse experiences in the implementation of PPP in health service delivery. However, this study only concentrated on PPP of health service delivery in one municipality (Kinondoni), of Dar es Salaam Region, and therefore findings may not reflect the experiences in other municipalities in Tanzania. Again, the study does not involve views from service users, mainly community members, who could have highlighted further challenges regarding the services in the consumers’ point of view. Despite the limitations, data from documents reviewed contributed towards bridging the gap in the methodological approach and empirical evidence.

## Conclusion

Challenges faced by PPP arrangements have centered on regulatory incompliance and laxity in enforcement, which culminates into poor implementation practices. Despite the challenges, provision of health services in Tanzania through public-private partnership, is still a viable alternative, given the existing resource constraints to construct public facilities and manage them. The public sector alone cannot solve the growing need for quality health services. Thus, the responsible authorities need to address these challenges by engaging private partners in all issues concerning their working contracts and consider partners as equal in implementing plans. Strengthening of communication between partners should be mandatory, since it is a strategy through which all stakeholders get to know the successes recorded, challenges experienced and the way forward. Communication channels should be improved to build trust, encourage transparency and accountability.

## Supplementary information

**Additional file 1.** Interview Guide.

## Data Availability

The datasets generated and/or analysed during the current study are not publicly available, but may be availed by the corresponding author on reasonable time request.

## Abbreviations

AIDS: Acquired immune deficiency syndrome
ARVs: Antiretroviral
BRN: Big Result Now
CHSB: Council for Health Service Board
CHMT: Council Health Management Team
CCHP: Comprehensive Council Health Plan
COSTECH: Commission for Science and Technology
DMO: District Medical Officer
HSR: Health Sector Reforms
HIV: Human immunodeficiency virus infection
HFGC: Health Facility Governing Committees
LGAs: Local Government Authorities
MCH: Maternal and Child Health Care
MoHCDGEC: Ministry of Health, Community Development, Gender, Elderly and Children
NHIF: National Health Insurance Fund
PPP: Public-Private Partnerships
SAPs: Structural Adjustment Programs
TB: Tuberculosis
WHO: World Health Organisation
