# Supplementary material for: Challenges in health service delivery under public-private partnership in Tanzania: stakeholders’ views from Dar es Salaam region
Source: BMC Health Serv Res. 2020 Aug 18;20:765. doi: 10.1186/s12913-020-05638-z (PMC7436953; doi:10.1186/s12913-020-05638-z)
Supplement: Supplementary file 1 — Additional file 1. Interview Guide. [file 12913_2020_5638_MOESM1_ESM.docx]

**File 1 Interview Guide**

**Interview Guide with Government Officials from the MoHCDGEC and Municipality**

**1. Participant profile**

Age (optional), sex (optional), educational qualification, professional training, designation, work experience

**2. Institutional arrangement**

1. Institutional arrangement in relation to role and mandate.
2. Existing mechanisms for ensuring effective institutional arrangement.
3. Procedure for applying for partnership contractual mechanism.

**3. Collaborations and relationships**

1. Areas of partnerships and the structure to ensure smooth provision of health service delivery.
2. Strategies to engage the private sector in health service delivery.

**4. Responsibilities**

1. Communication channels and information flow between partners.
2. Managing and implementing PPP policies, regulations and strategies.
3. Partners responsibilities in the PPP framework in regard to planning, budgeting and implementation.
4. Capacity building strategies and initiatives.
5. Monitoring and evaluation mechanisms under PPP.
6. Roles of existing bodies guiding PPP.

**5. Benefits of PPP**

1. Level of improvement in provision of health services.
2. Current and potential future benefits from PPP arrangements.

**6. Challenges under PPP and way forward**

1. Common challenges experienced.
2. Proposed solutions in managing and implementing PPP policies and strategies.

**Interview guide with officials from health facilities under PPP**

**1. Participant profile**

Age (optional), sex (optional), educational qualification, professional training, designation, work experience

**2. Collaboration and relationship**

1. The status and nature of collaboration and relationship.
2. Contractual mechanisms and mode of agreement.
3. Funding mechanisms and capacity building opportunities.

**3. Roles and responsibilities**

1. Health facility partnership activities.
2. Accountability processes and mechanisms.
3. Communication and information sharing structures and systems.
4. Participation in planning and budgeting activities.
5. Participation in decision making meetings with bodies guiding PPP.
6. Monitoring and evaluation mechanisms.

**4. Accrued benefits in the participation of PPP**

1. Technical.
2. Financial.

**5. Challenges under PPP and way forward**

1. Significant challenges hindering service provision under PPP.
2. Recommendations and suggestions.
